# Clinical phenotype and genetic function analysis of a family with hypomyelinating leukodystrophy-7 caused by *POLR3A* mutation

**DOI:** 10.1038/s41598-024-58452-6

**Published:** 2024-04-01

**Authors:** Dan-dan Ruan, Xing-lin Ruan, Ruo‑li Wang, Xin-fu Lin, Yan-ping Zhang, Bin Lin, Shi-jie Li, Min Wu, Qian Chen, Jian-hui Zhang, Qiong Cheng, Yi-wu Zhang, Fan Lin, Jie-wei Luo, Zheng Zheng, Yun-fei Li

**Affiliations:** 1 Department of Traditional Chinese Medicine, Shengli Clinical Medical College of Fujian Medical University, Fujian Provincial Hospital, Fuzhou, China; 2 Department of Neurology, Fujian Medical University Union Hospital, Fuzhou, 350001 China; 3 Fujian Provincial Key Laboratory of Emergency Medicine, Fujian Provincial Institute of Emergency Medicine, Fujian Emergency Medical Center, Fuzhou, 350001 China; 4 Pediatrics Department, Fujian Provincial Hospital, Fuzhou, 350001 China; 5 Department of Neurology, Fujian Provincial Hospital, Fuzhou, 350001 China; 6 Department of Neurology, Youxi County General Hospital, Sanming, 365100 China; 7 Department of Geriatric Medicine, Fujian Provincial Center for Geriatrics, Fujian Provincial Hospital, Fuzhou, 350001 China

**Keywords:** POLR3-related hypomyelinating leukodystrophy, POLR3A gene, HLD-7, RNA polymerase III (Pol III), Myelination, Neuroscience, Neurology, Clinical genetics, Gene expression, Gene regulation, Genetic association study, Inbreeding, Neurodevelopmental disorders

## Abstract

Hypomyelinating leukodystrophy (HLD) is a rare genetic heterogeneous disease that can affect myelin development in the central nervous system. This study aims to analyze the clinical phenotype and genetic function of a family with HLD-7 caused by *POLR3A* mutation. The proband (IV6) in this family mainly showed progressive cognitive decline, dentin dysplasia, and hypogonadotropic hypogonadism. Her three old brothers (IV1, IV2, and IV4) also had different degrees of ataxia, dystonia, or dysarthria besides the aforementioned manifestations. Their brain magnetic resonance imaging showed bilateral periventricular white matter atrophy, brain atrophy, and corpus callosum atrophy and thinning. The proband and her two living brothers (IV2 and IV4) were detected to carry a homozygous mutation of the *POLR3A* (NM_007055.4) gene c. 2300G > T (p.Cys767Phe), and her consanguineous married parents (III1 and III2) were p.Cys767Phe heterozygous carriers. In the constructed *POLR3A* wild-type and p.Cys767Phe mutant cells, it was seen that overexpression of wild-type POLR3A protein significantly enhanced Pol III transcription of 5S rRNA and tRNA Leu-CAA. However, although the mutant POLR3A protein overexpression was increased compared to the wild-type protein overexpression, it did not show the expected further enhancement of Pol III function. On the contrary, Pol III transcription function was frustrated (*POLR3A*, *BC200*, and tRNA Leu-CAA expression decreased), and *MBP* and 18S rRNA expressions were decreased. This study indicates that the *POLR3A* p.Cys767Phe variant caused increased expression of mutated POLR3A protein and abnormal expression of Pol III transcripts, and the mutant POLR3A protein function was abnormal.

## Introduction

Leukodystrophy is a group of heterogeneous neurodegenerative diseases characterized by abnormal white matter imaging^1^. They are classified as hypomyelinating leukodystrophy (HLD) and non-hypomyelinating leukodystrophy based on white matter abnormalities on magnetic resonance imaging (MRI) of the brain^2^. POLR3-related hypomyelinating leukodystrophy (POLR3-HLD) is an autosomal recessive hypomyelinating leukodystrophy featured by neurological and non-neurological systems, and its typical clinical phenotype includes hypomyelination, hypodontia, and hypogonadotropic hypogonadism, therefore it is also known as 4H syndrome^3^. POLR3-HLD is caused by mutations in the *POLR3A*, *POLR3B*, *POLR1C*, and *POLR3K* genes, which encode subunits of human RNA polymerase III (Pol III)^4^. Pol III is the largest of the three RNA polymerases, containing 17 subunits. *POLR3A* encodes the largest subunit of Pol III, which together with POLR3B forms the catalytic center of the enzyme. Pol III is involved in the transcription of a variety of small molecules of non-coding RNAs (NC-RNAs), including transfer RNAs (tRNAs), 5S ribosomal RNA (rRNA), 7SL and 7SK RNAs, and U6 small nuclear RNA^5^. Most Pol III transcripts are involved in the regulation of essential processes such as transcriptional regulation, RNA processing, and protein translation^6^, and the normal function of Pol III plays a key role in the maintenance and development of myelin^7^. In addition, Pol III-transcribed brain cytoplasmic 200 RNA (BC200 RNA) is highly expressed in the central nervous system (CNS), so its role in neurological disorders deserves further investigation. BC200 RNA is a primate brain-specific non-messenger RNA^8^, which is considered to be a translational regulator in the synaptic-dendritic structural domain of neurons and is associated with the regulation of dendritic local protein synthesis^9^. However, the role of BC200 RNA in myelin formation is rarely studied.

The impairment of Pol III or tRNA function can easily affect the CNS. A variety of neurological diseases, including several leukodystrophy, have been found to result from mutations in genes associated with tRNA biology^10–13^. *POLR3A* gene is constitutively expressed in all tissues, and although it is not expressed at very high levels, it is the highest in the cerebellum^14^. *POLR3A* mutation may lead to dysfunction of Pol III, which affects the expression of certain tRNAs that are more abundant in the CNS, and thus disrupts protein synthesis^15^. In our previous work, we identified a hypomyelinating leukodystrophy-7 (HLD-7) family and detected the homozygous variation of *POLR3A* (NM_007055.4) gene c. 2300G > T (p.Cys767Phe). At present, there is no literature to study the function of the mutation site, and its clinical significance is unclear. Therefore, this study intends to analyze the clinical phenotype of the family and study the function of the mutation site to further investigate the pathogenicity of this mutation. It is expected that this study can improve clinicians’ understanding of the HLD-7 and POLR3-HLD disease spectrum and provide a reference for clinical diagnosis and treatment of this disease.

## Materials and methods

### Research object

**Figure 1:**
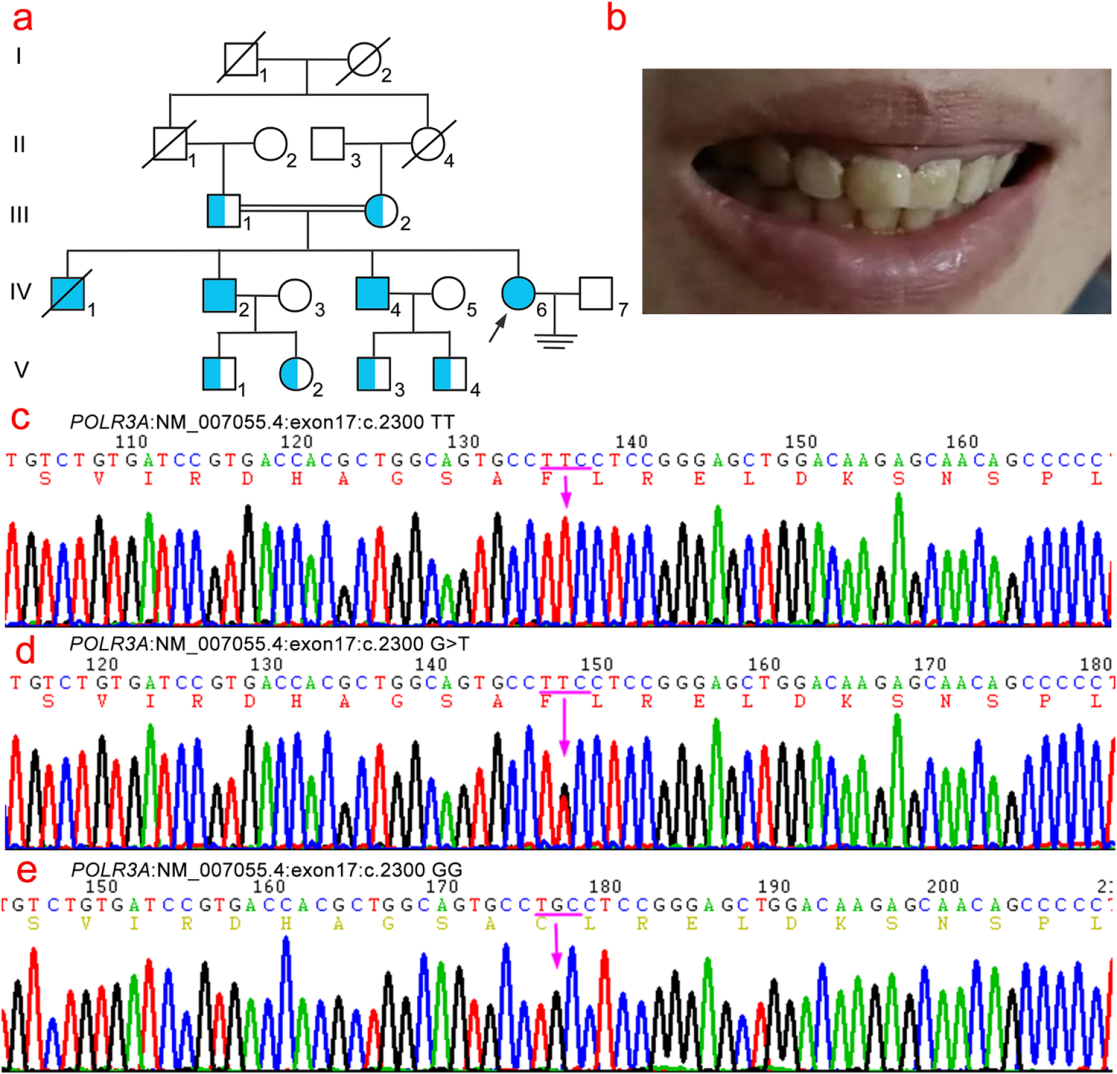
Genogram and Sanger sequencing diagram of the family. (**a**) Pedigree diagram of a family with hypomyelinating leukodystrophy-7, in which III1 is also complicated by Finnish type renal amyloidosis; the proband (arrow) carries a homozygous mutation of *POLR3A* (NM_007055.4) c.2300G > T (p.Cys767Phe); blue indicates carrying *POLR3A* (NM_007055.4) c.2300G > T (p.Cys767Phe) variant. (**b**) The proband had dentin dysplasia. (**c**) c.2300G > T (p.Cys767Phe) homozygous mutant at exon 17 of *POLR3A* gene. (**d**) c.2300G > T (p.Cys767Phe) heterozygous mutant at exon 17 of *POLR3A* gene. (**e**) Sanger sequencing verification map of the wild-type *POLR3A* gene.

The proband (IV6, Fig. 1a), a 36-year-old female, presented to our hospital with the chief complaint of “dizziness and memory decline for 16 years”. Collect the relevant medical history and clinical data of the proband and other members of the family, such as biochemistry, serum ceruloplasmin, thyroid function including thyroid stimulating hormone (TSH), free triiodothyronine (FT3), and free thyroxine (FT4), parathyroid hormone, and sex hormone (follicle-stimulating hormone [FSH], luteinizing hormone [LH], estradiol [E2], progesterone [P4], testosterone [T], prolactin [PRL]). The results of breast color ultrasound, vaginal color ultrasound, brain MRI, electroencephalogram (EEG), electromyogram (EMG), blink reflex, somatosensory evoked potential (SEP), and event-related evoked potential P300 of the proband were collected. This study was approved by the Ethics Committee of Fujian Provincial Hospital, China, and all the family members participating in this study signed informed consent forms.

### Extraction of genomic DNA

Peripheral blood of the proband and family members were collected with an ethylenediaminetetraacetic acid anticoagulant tube, and genomic DNA of the proband and family members were extracted according to the instructions of the QIAamp DNA Blood Mini Kit (QIAGEN Co., Ltd.). The extracted DNA samples were examined using the NanoDrop instrument, agarose gel electrophoresis, and Qubit, and used for the subsequent sequencing process.

### Whole-exome sequencing and variant screening and interpretation

Next-generation sequencing (NGS) was performed on the proband. Break genomic DNA and use a reagent kit for end-repaired, adding adenine bases, and connecting connectors, the genomic DNA library was obtained after polymerase chain reaction (PCR) amplification and PCR product purification. NEXome Core Panel and Hybridization and Wash kits were used for a whole-exome capture in the library, and the quality of the library was tested after PCR linear amplification. The coverage of the target area is 99.63%, and the average sequencing depth of the target area is 160.88 ×. After passing the standard, PE 150 sequencing was performed using a DNBSEQ-T7 sequencer. The capture target genes involved include *NOTCH3*, *POLR3A*, *POLR3B*, *POLR3K*, *POLR1C*, *CYP7B1*, and *MADD*. The sequenced fragments were compared with the UCSC hg19 human reference genome by BWA software, and the data were sorted and de-duplicated using Picard and Samtools markdup software, then the data were analyzed for single nucleotide variant and insertion and deletion using GATK software, and copy number variation at the exon level was detected using ExomeDepth. Combined with the data of mutation detection, the phenotypic-related sites were analyzed by Exomiser software, and the suspicious mutations were screened and interpreted according to the American College of Medical Genetics and Genomics guidelines. Primer 5.0 was used to design specific primers for the upstream and downstream positions of the target mutation site sequence, and the target region was amplified. Sanger sequencing was performed using an ABI 3730XL DNA sequencer (ABL, USA). The amplified fragment length of the target sequence of c. 2300G > T (p.Cys767Phe) of *POLR3A* (NM_007055.4) was 800 bp, and the primer was F: TGAGGGCCTCGGTATCTACAA; R: TGATCGGAAATCGGGCATCT. The annealing temperature was 60 °C. The primers were synthesized by Beijing liuhe BGI Technology Co., Ltd.

### Plasmid construction and identification

The coding sequences (CDS) of *POLR3A* (NM_007055.4) and *POLR3A* p.Cys767Phe mutant were constructed into pCDH-CMV-MCS-EF1-copGFP-T2A-puro vector, XbaI/NotI was selected as the double digestion site, and the N-terminal end of the CDS carried the FLAG tag. The target gene was amplified and verified by sequencing. The plasmid vector construction and related PCR primer synthesis were done with the assistance of Wuhan gene create Co., Ltd (Wuhan, China).

### Cell culture and transfection

The hela cells were placed in 1640 complete culture medium containing 1% Penicillin–Streptomycin Solution and 10% Fetal bovine serum and cultured at 37 °C in a 5% CO2 incubator, and the cells were routinely passaged at approximately 90% cell density. The hela cells were digested and collected by trypsin, and the cells were laid on a 6 cm cell-culture dish at a density of 5 × 10^5^ cells/plate in an appropriate complete medium. The cells were incubated overnight in a 37 °C incubator with 5% CO2 according to the cell adherence, and transient transfection could be started after the cells were fully adherent. Prepare TurboFect-DNA Mix according to TurboFect instructions, add 6 µg of plasmid and 12 µL of TurboFect into 600 µL of Opti-Medium and mix gently, and then incubated at room temperature for 15 min. Add TurboFect-DNA Mix drop by drop to the cell culture medium of the above monolayer cells, shake the dish gently and mix well, and incubate in a 37 °C incubator containing 5% CO2. After 8–12 h of incubation, the medium was replaced with serum-containing medium preheated at 37 °C, and after continuing the culture up to 48 h, cell samples were collected for subsequent verification of cell function.

### Real-time quantitative polymerase chain reaction (RT-qPCR)

The cells were collected for RNA extraction, reverse transcription, and qRT detection. Primers were designed with Primer 5.0 (Table 1).

**Table 1:** Primers used in real-time quantitative polymerase chain reaction.

Primer name	Primer sequence (5′-3)
hPOLR3A qRT F	GCTCGACCATAGGATGGGTA
hPOLR3A qRT R	TTCTCCTCTTGGGACAGCAT
MBP qRT F	CTCCCAAGGCACAGAGACAC
MBP qRT R	GGAGCCGTAGTGAGCAGTTC
BC200 qRT F	GCCTGTAATCCCAGCTCTCA
BC200 qRT R	GGTTGTTGCTTTGAGGGAAG
hGAPDH F	CAAGGTCATCCATGACAACTTTG
hGAPDH R	GTCCACCACCCTGTTGCTGTAG
5S rRNA qRT F	GCCATACCACCCTGAACG
5S rRNA qRT R	AGCCTACAGCACCCGGTATT
tRNA Leu-CAA qRT F	CTCAAGCTTGGCTTCCTCGT
tRNA Leu-CAA qRT R	GAACCCACGCCTCCATTG
7SK RNA qRT F	AGAGGACGACCATCCCCGAT
7SK RNA qRT R	TGGAAGCTTGACTACCCTACGT
18S rRNA qRT F	GTAACCCGTTGAACCCCATT
18S rRNA qRT R	CCATCCAATCGGTAGTAGCG
28S rRNA qRT F	AGAGGTAAACGGGTGGGGTC
28S rRNA qRT R	GGGGTCGGGAGGAACGG

### Cell immunofluorescence

After being fixed, transparent, and blocked, the transfected hela cells were incubated overnight at 4 °C with the Flag primary antibody (Anti-FLAG 1:1000). The transfected hela cells were rinsed with phosphate-buffered saline (PBS) three times; the fluorescent secondary antibody (Goat Anti-Mouse, final concentration 2 μg/mL) was added and incubated at room temperature in the dark for 2 h, rinsed 3 times with PBS; stained with 4′,6-diamidino-2-phenylindole and incubated at room temperature for 10 min, rinsed 3 times with PBS; sealed with 50% glycerol; and photographed by laser confocal microscopy after sealing.

### Western Blot

The hela cells were cultured and lysed, the total protein was extracted, and the protein concentration was detected with a multifunctional enzyme marking instrument. After the electrophoresis device was set up, protein samples, as well as protein Maker, were added to the wells on the electrophoresis gel in the desired order using a pipette or a loading needle. After electrophoresis, the proteins were wet transferred to the polyvinylidene fluoride (PVDF) membrane. The membrane after protein transfer was rinsed with Tris-buffered saline with 0.1% Tween® 20 (TBST) and sealed at room temperature for 1 h by adding 5% bovine serum albumin (BSA) blocking solution per 0.1 mL/cm^2^. After sealing, the PVDF membrane was rinsed with TBST once. The primary antibody (Anti-FLAG, 1:2000; Anti-α-Tubulin, 1:1000) was diluted with 5% BSA to the appropriate concentration, put into PVDF membrane and react at 37 °C for 1 h or overnight at 4 °C; after washing the membrane 3 times, add the secondary antibody (Goat Anti-Mouse) labeled with horseradish peroxidase diluted with 5% BSA, and put it into the PVDF membrane and react at room temperature for 1 h; wash for 5 times and then expose.

### Statistics

The data of 2^−△△CT^ values obtained by RT-qPCR were statistically analyzed by GraphPad Prism version 6.0 software. The measurement data were expressed as mean ± standard error of the mean. A one-way analysis of variance was used for comparison between multiple groups. The least significant difference test was used for comparison among groups.

### Ethical approval

All procedures were performed in accordance with the tenets of the Declaration of Helsinki and the study was approved by the Ethics Committee of Fujian Provincial Hospital, Fuzhou, China. All participants and legal guardians of the minors involved in the present study provided written informed consent.

## Results

### Clinical phenotypes

**Figure 2:**
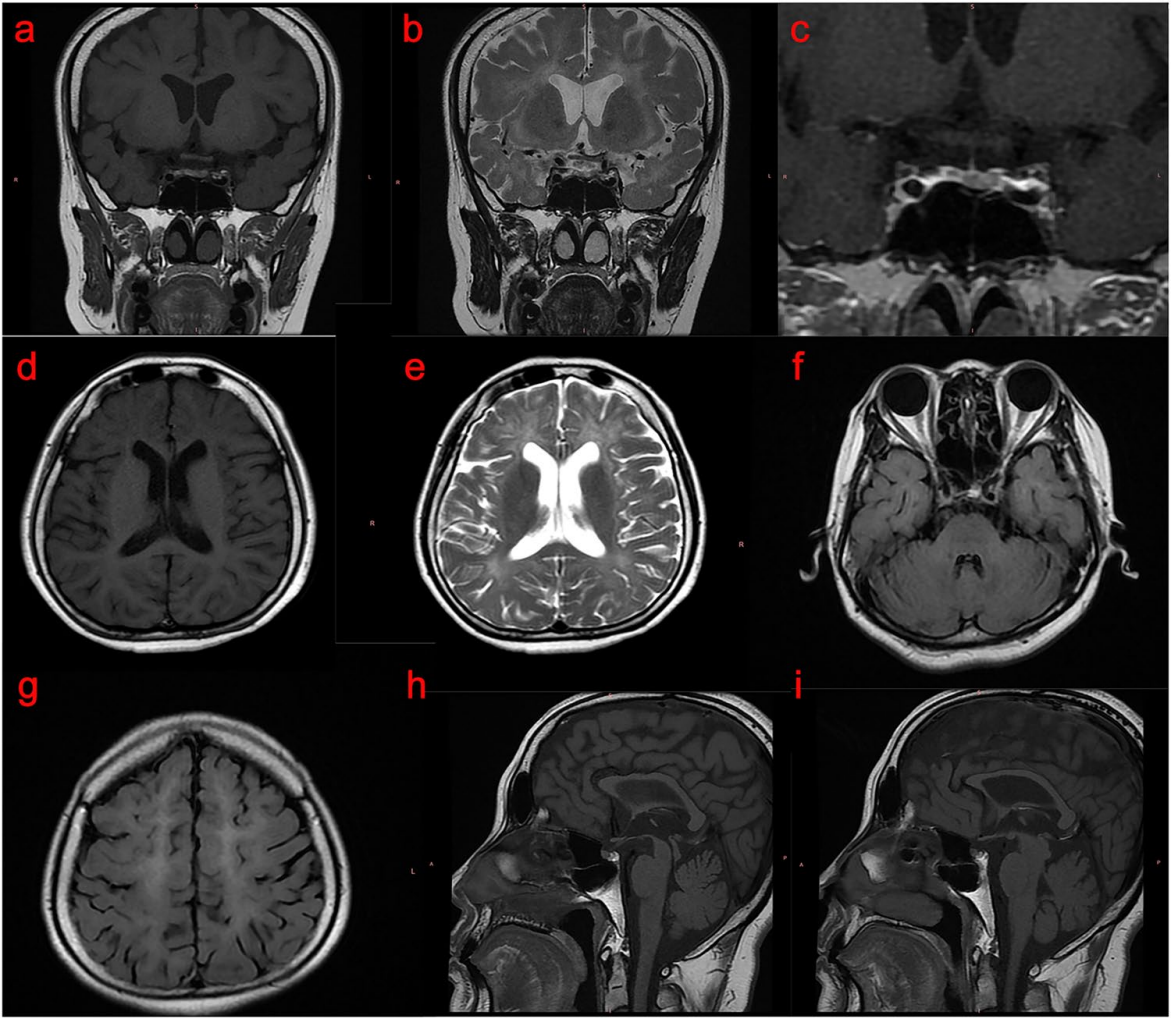
Craniocerebral magnetic resonance imaging (MRI) results of the proband. (**a**–**i**) Craniocerebral MRI showed that the pituitary gland was approximately 3.8 mm in height, with a flat upper edge and a slightly sunken saddle base, and multiple tiny nodules with equal and slightly long T1 signals and slightly long T2 signals were seen inside, and the dynamic enhancement showed relatively slightly low signals, the larger one being approximately 2.2 mm in diameter; bilateral periventricular white matter atrophy and degeneration, with short linear and patchy equal and slightly long T1 and slightly long T2 signals, and slightly high T2-Flair signal; cerebral sulcus, cistern, and ventricle were generally widened; corpus callosum atrophy and thinning, particularly at the knee and body of the corpus callosum.

The proband (IV6) is a 36-year-old woman. The proband had paroxysmal dizziness and memory decline at the age of 20, manifested by frequent forgetfulness, accompanied by rapid temper, but her life and work had not been significantly affected. Brain MRI in the local hospital showed white matter degeneration and cerebellar atrophy. Urine routine indicates microalbuminuria. No headache, limb numbness, fever, nausea, vomiting, speech confusion, and no dyskinesia. There was a history of delayed eruption of deciduous teeth and infertility. She has no history of drinking, taking drugs, and taking phenothiazines, no history of psychosis, encephalitis, brain trauma, and stroke, and no history of adverse reactions to vaccines. The proband had menarche at the age of 17 and had irregular menstruation, requiring long-term oral estradiol tablets/estradioland dydrogesterone tablets. Married at the age of 34, currently infertile. Her parents (III1 and III 2) were consanguineous marriage and had no similar medical history, but her three older brothers (IV1, IV2, and IV4) all had a history of cognitive dysfunction. Physical examination: height 167 cm, weight 60 kg, BMI 21.26 kg/m^2^, good nutritional status, normal blood pressure in both prone and standing positions, no K-F ring in the cornea, dentin dysplasia (Fig. 1b), no significant abnormalities in heart, lung, and abdominal examination. Breast and pubes development stagnated at Tanner Stage II, vulvar development was immature, and there were no deformities in spine and limb development. Nervous system physical examination: clear consciousness, clear speech, answering questions correctly, cooperative examination; memory slightly decreased, MMSE 27 points, MoCA 15 points (bachelor degree). The sense of smell was normal, and the cranial nerve examination was normal. The muscle strength, muscle tension, and tendon reflex of the limbs were normal. Bilateral superficial and deep sensation existed symmetrically. The movement was flexible, and the gait was normal. The bilateral finger-nose test was accurate, the alternating movement test was coordinated, the heel-knee-tibia test was normal, and Romberg’s sign was negative. Bilateral pathological signs were negative. Auxiliary examination: Thyroid function examination in other hospitals showed that TSH 0.92 (0.27 ~ 4.20) uIU/ml, FT3 5.87 (3.1 ~ 6.8) pmol/L, and FT4 16.9 (12 ~ 22) pmol/L; anti-mullerian hormone 5.24 (0.777 ~ 5.24) ng/ml; repeated monitoring of sex hormone levels showed E2 13.7 ~ 26 (49 ~ 291) pg/ml, FSH 0.05 ~ 0.17 (1.79 ~ 5.12) mIU/ml, LH 0.01 (1.20 ~ 12.86) mIU/ml, PRL 2.06 ~ 3.7 (3.34 ~ 26.72) ng/ml, P4 0.29 ~ 0.55 (5.16 ~ 18.56) ng/ml, and T 0.35 ~ 0.46 (0.1 ~ 0.75) ng/ml. At that time, the gonadotropin-releasing hormone (GnRH) stimulation test showed that LH and FSH had no significant response. Breast color ultrasound showed that the structure of the glandular layer was disordered and the arrangement was not clear. Vaginal ultrasound shows the posterior position of the uterus, with a clear contour and regular shape, approximately 42 mm × 39 mm × 27 mm in size; its muscular layer has uniform echoes and no obvious abnormal mass echoes was observed. After admission, a craniocerebral MRI showed multiple tiny nodular foci in the pituitary gland, considering microadenoma; hypomyelination of the white matter of the brain; brain atrophy; and atrophy and thinning of the corpus callosum (Fig. 2). EEG, EMG, and blink reflex tests showed no significant abnormalities (the detailed results of EMG can be found in Supplementary Table S1). SEP: both upper limbs were normal, the left lower limb was abnormal, and the right lower limb was normal (see Supplementary Table S2). P300 detection: P300 waveform differentiation and repeatability were acceptable, the latency was slightly prolonged and the reaction time was prolonged (Table 2). No obvious abnormality was found in biochemistry and serum ceruloplasmin.

**Table 2:** Clinical characteristics of the patients in the family with hypomyelinating leukodystrophy-7.

Subject	IV6	IV1	IV2	IV4
Gender	Female	Male	Male	Male
Age (years)	36	Deceased	39	38
Height (cm)	167	180	184	180
POLR3A p.Cys767Phe	Hom	Unknown	Hom	Hom
Age of onset of neurological symptoms (years)	20	16	20	23
Cognitive dysfunction	Mild	Moderate to severe	Moderate to severe	Severe
Puberty development	Delayed, stagnant	Delayed	Delayed	Delayed
Hypogonadotropic hypogonadism	Yes	Unknown	No	No
Abnormal tooth development	Delayed eruption of deciduous teeth and dentin dysplasia	Delayed eruption of permanent teeth and lack of the right maxillary median incisor	Delayed eruption of permanent teeth	Delayed eruption of deciduous teeth and lack of the left maxillary canine
Hair and nails	Pubes development stagnated at Tanner Stage II with normal nails	Unknown	Normal	Normal hair development, but nails partially missing
Ataxia	No	Yes	Yes	Yes
Dysarthria	No	Yes	No	Yes
Dystonia	No	Yes	No	Yes
Muscle atrophy of extremities	No	Unknown	Mild	Moderate to severe
Extremity muscle strength	Normal	Unknown	Muscle strength of both lower limbs is grade 4	Extremity muscle strength grade 3
Tendon reflexes of extremities	Normal	Unknown	Bilateral upper limbs ( +), bilateral lower limbs (+ + +)	Bilateral upper limbs ( +), bilateral lower limbs (+ + +)
Bilateral superficial and deep sensation	Normal	Unknown	ND	ND
Bilateral pathological signs	Negative	Unknown	Positive ( +)	Positive ( +)
EMG	Normal	Unknown	ND	ND
Latency of blink reflex R1/R2/R2’ (ms)	Left stimulus: 11.3/34.2/34.5; Right stimulus: 12.2/34.5/35.0	Unknown	ND	ND
SEP	Bilateral upper limbs normal; left lower limb abnormal, right lower limb normal	Unknown	ND	ND
P300 latency Fz/Cz/Pz (ms)	398/416/436	Unknown	454/453/401	No definite waveform
P300 amplitude Fz/Cz/Pz (mV)	487/536/533	Unknown	243/243/260	No definite waveform
Wheel-chair use	No	Yes	No	Yes
Age at death (years)	N/A	38	N/A	N/A

The craniocerebral magnetic resonance imaging of patients in this family with hypomyelinating leukodystrophy-7 (HLD-7) all showed symmetrical white matter abnormalities around the ventricles, corpus callosum thinning, and cerebellar atrophy. Patients with HLD-7 all showed psychiatric symptoms such as euphoria, anxiety disorders, and emotional instability at an early stage of the disease. This family has not presented with symptoms of optic atrophy, epilepsy, or postural tremor. The detailed results of IV6’s EMG and SEP can be found as Supplementary Tables S1 and S2 online. Hom, homozygote; EMG, electromyography; SEP, somatosensory evoked potential; ND, not done; N/A, not applicable.

The proband’s eldest brother (IV1), height 180 cm, delayed puberty, unmarried and infertile. IV1 had delayed eruption of permanent teeth and lack of the right maxillary median incisor. Neurological symptoms appeared at the age of 16, mainly characterized by personality changes, emotional instability, memory loss, slurred speech, and progressive walking difficulties. He began to use a wheelchair at the age of 21 because he was unable to stand. He became bedridden around the age of 30 and has been dead for 3 years (age of death 38). The proband’s second brother (IV2) was 39 years old, height 184 cm, and had a son and a daughter. His children are all healthy. IV2 had delayed eruption of permanent teeth, delayed puberty, and no significant abnormalities in sex steroid levels. At the age of 20, he began to experience personality changes, manifesting as euphoria, exaggerated delusions, and gradually developing memory loss and unstable walking, and currently can still walk independently. Physical examination: there was no obvious abnormality in the general examination and physical examination of the heart, chest, and abdomen. He was clearly conscious, could answer correctly, and was more euphoric, but was uncooperative in the intelligence examination and could not complete the clock drawing test. The cranial nerve examination was negative, the muscle tension of the extremities was normal, the muscle strength of both upper limbs was grade 5, and the muscle strength of both lower limbs was grade 4. Mild atrophy of extremity muscles. The bilateral finger-nose test was normal, the heel-knee-tibia test was uncooperative, the walking straight line was not possible, Romberg’s sign was positive, and bilateral superficial and deep sensory examination was uncooperative. Tendon reflexes of both upper limbs (+), tendon reflexes of both lower limbs (+ + +). Patellar clonus (−), bilateral ankle clonus (+), and bilateral pathological signs (+). P300 detection: P300 waveform differentiation and repeatability were poor, with prolonged latency and reaction time (Table 2). The proband’s third brother (IV4), 38 years old, height 180 cm, and had 2 sons. His sons were both healthy. IV4 had delayed eruption of deciduous teeth and lack of the left maxillary canine; puberty was delayed but sex steroid levels were not significantly abnormal. He started to experience neurological symptoms at the age of 23, first manifesting as excessive speech and was treated with oral olanzapine in an external psychiatric unit (details unknown). Thereafter, he gradually developed decreased speech, dullness, and memory loss. He developed progressive unsteadiness in walking at the age of 30 and started using a wheelchair at the age of 35 due to inability to stand. Physical examination: partial cooperation on physical examination, malnourished appearance, and sent to hospital in a wheelchair. Cardiac, thoracic, and abdominal examinations showed no abnormalities. He was clearly conscious, with a dull expression and markedly decreased reaction speed, and was unable to complete the clock drawing test. Difficulty in eye tracking and scanning, with bilateral nasolabial folds symmetrical in a natural state, and tongue extension in the center; minimal speech, dysarthria, and the remaining cranial nerve examination were uncooperative. The muscle tension of both lower limbs was increased, and the muscle strength of the extremities was grade 3. Moderate-to-severe atrophy of extremity muscles. The ataxic motor and bilateral superficial and deep sensory examinations were uncooperative. Tendon reflexes of both upper limbs (+), tendon reflexes of both lower limbs (+ + +), patellar clonus (−), bilateral ankle clonus (+), and bilateral pathological signs (+). P300 detection: no definite P300 wave was observed. The craniocerebral MRI of the three old brothers of the proband all showed symmetrical white matter abnormalities around the ventricles, corpus callosum thinning, and cerebellar atrophy. Unfortunately, except for P300, IV2 and IV4 did not cooperate with EMG and SEP testing, so it was difficult to distinguish whether their extremity muscle atrophy was wasting atrophy or atrophy due to peripheral neuropathy. All members of this family also refused to undergo nerve biopsy. In addition, the father of the proband had manifestations of proteinuria and nephritis, and is currently undergoing hemodialysis in their local hospital due to uremia. He was diagnosed with Finnish type renal amyloidosis by gene sequencing in our department. The clinical features of the proband and family members were summarized in Table 2.

### Screening for mutated genes

Through the analysis of NGS and exon capture techniques, a homozygous mutation c. 2300G > T (p.Cys767Phe) in exon 17 of *POLR3A* (NM_007055.4) was found in the proband (IV6), which led to the conversion of cysteine (Cys) to phenylalanine (Phe) at position 767 of POLR3A protein. The proband’s two surviving brothers (IV2 and IV4) also carried a homozygous mutation of *POLR3A* (p.Cys767Phe), and their consanguineous married parents (III1 and III2) carried a heterozygous mutation of *POLR3A* (p.Cys767Phe). In addition, V1, V2, V3, and V4 also carried the *POLR3A* (p.Cys767Phe) heterozygous mutation (Fig. 1c–e).

### Cloning of *POLR3A* wild-type and p.Cys767Phe mutant

**Figure 3:**
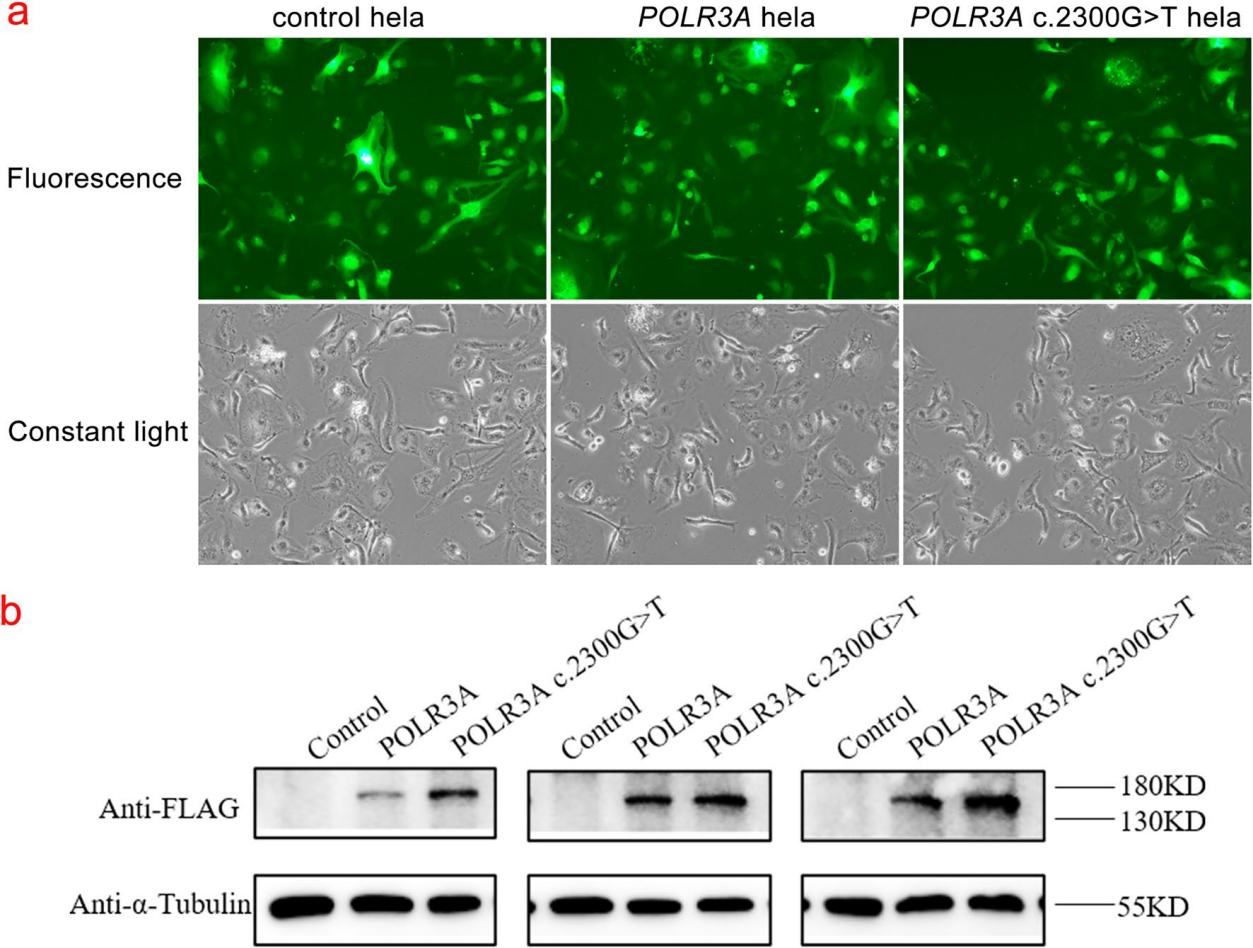
The situation of cell culture and the detection results of Western Blot (WB). a. Basal culture growth of hela cells after transfection. b. The expression of exogenous POLR3A protein in the cells was detected by WB. Original blots/gels are presented in Supplementary Fig. S1.

The pCDH-CMV- POLR3A-EF1-copGFP-T2A-Puro, pCDH-CMV- POLR3A c.2300G > T-EF1-copGFP-T2A-Puro, and control were transiently transfected. The fluorescence and growth of cells showed that the cells were in good condition (Fig. 3a).

### Expression of *POLR3A* wild-type and p.Cys767Phe mutant in hela cells

**Figure 4:**
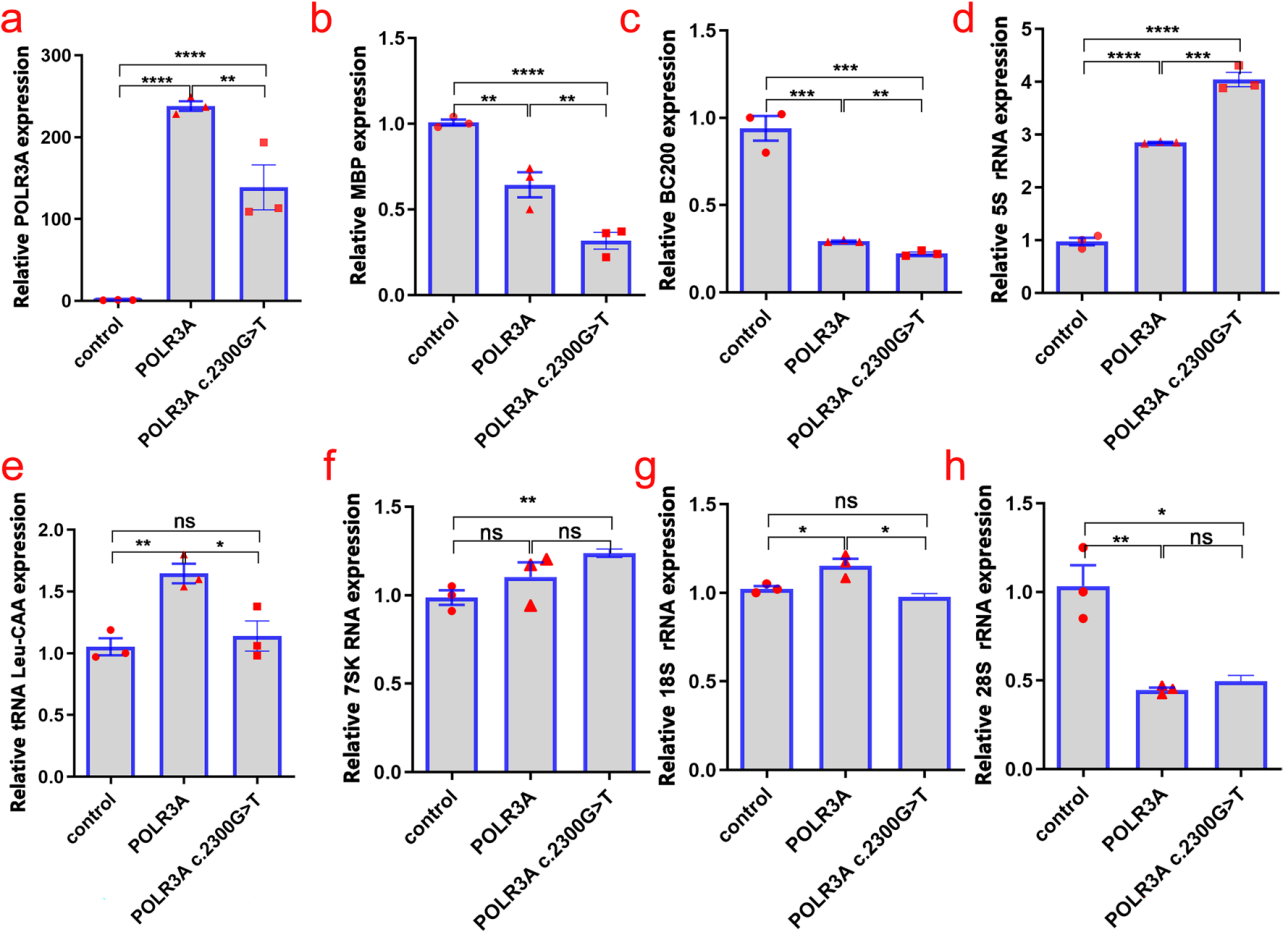
Expression levels of Pol III transcripts. The relative expression of *POLR3A*, *MBP*, *BC200*, 5S rRNA, tRNA Leu-CAA, 7SK RNA, 18S rRNA, 28S rRNA, and internal reference were detected by Real-time quantitative polymerase chain reaction. Data are presented as mean ± standard error of the mean, n = 3; ns, not significant, *P < 0.05, **P < 0.01, ***P < 0.001, ****P < 0.0001.

The expression of exogenous POLR3A protein in the cells was detected by WB (Fig. 3b). The results showed that compared with the control group, the expression of POLR3A protein increased after overexpression of both *POLR3A* wild type and *POLR3A* c. 2300G > T mutants, and the expression of POLR3A protein was higher than that of the wild type after *POLR3A* c. 2300G > T mutation. The expression of *POLR3A*, *MBP*, *BC200*, 5S rRNA, tRNA Leu-CAA, 7SK RNA, 18S rRNA, 28S rRNA, and internal reference in cells was detected by RT-qPCR (Fig. 4). The results showed that the overexpression of wild-type POLR3A protein could significantly enhance Pol III transcription of 5S rRNA, tRNA Leu-CAA. However, although the mutant POLR3A protein overexpression was increased compared to the wild-type protein overexpression, it did not show the expected further enhancement of Pol III function. On the contrary, Pol III transcription function was frustrated (*POLR3A*, *BC200*, and tRNA Leu-CAA expression decreased), and the expression of *MBP* and 18S rRNA was decreased, suggesting that the mutant POLR3A protein function was abnormal.

### Localization of POLR3A wild-type and p.Cys767Phe mutant in cells

**Figure 5:**
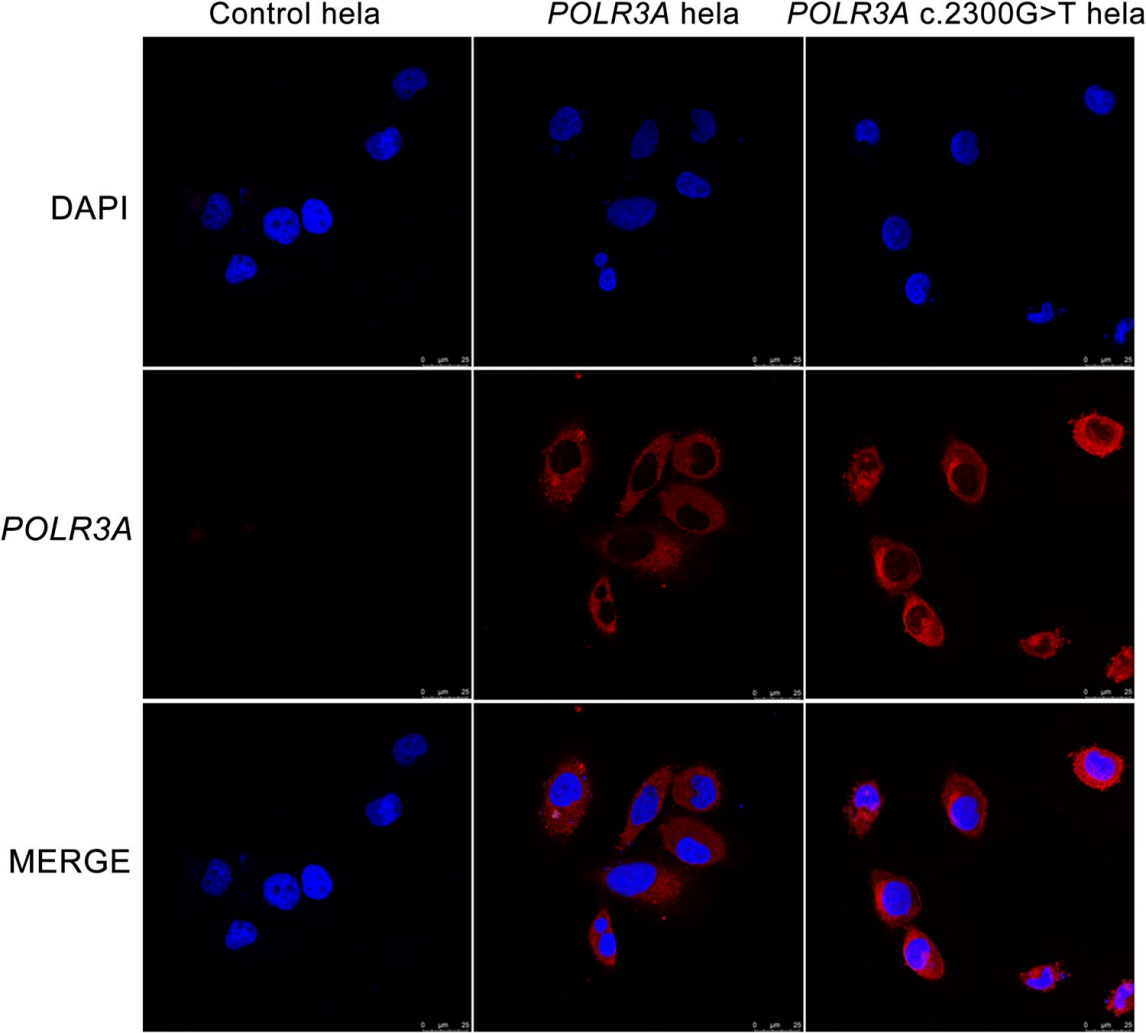
The expression and localization of POLR3A protein in hela cells. The wild-type POLR3A protein and POLR3A c.2300G > T mutant protein were uniformly distributed in the cytoplasm, and the expression of POLR3A protein was enhanced after *POLR3A* c. 2300G > T mutation.

Immunofluorescence was used to detect the expression and localization of the POLR3A protein (Fig. 5). Considering the effect of endogenous POLR3A, this part of the experiment was performed using Flag antibody, and the results showed that wild-type POLR3A protein and POLR3A c.2300G > T mutant protein were uniformly distributed in the cytoplasm, and the expression of POLR3A protein was enhanced after *POLR3A* c. 2300G > T mutation.

### Bioinformatics prediction of proteins

**Figure 6:**
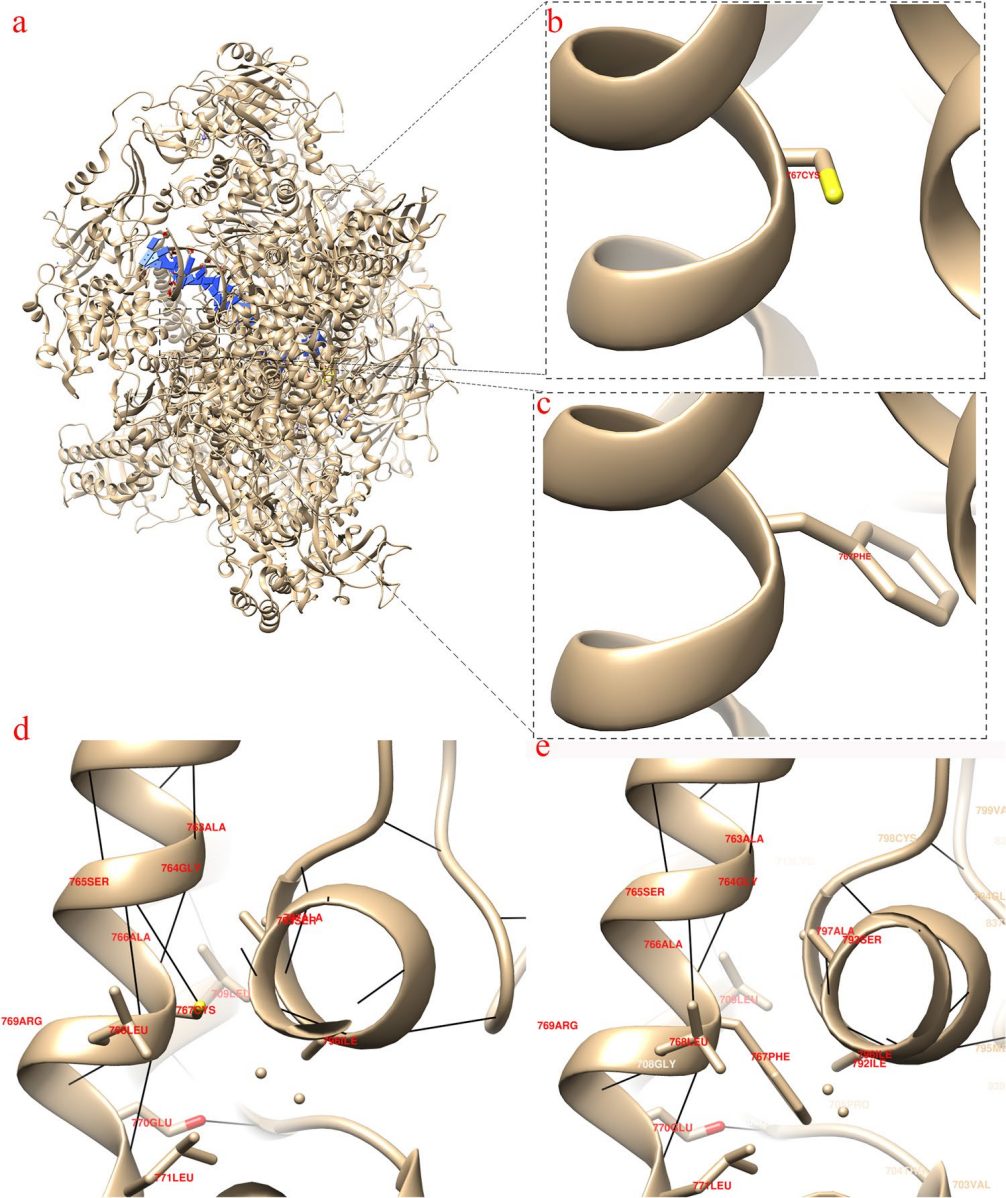
Structure prediction of the POLR3A protein. a. Three-dimensional structure of POLR3A protein. b. The local base structure of Cys at position 767. c. The local base structure of mutation to 767 Phe. d. Local structure diagram of 767 Cys with adjacent amino acids. e. Local structure diagram of 767 Phe with adjacent amino acids. The black lines represent hydrogen bonds.

The protein structure of POLR3A was searched at https://www.rcsb.org/sequence/7AE1, and the mutated protein structure was predicted by Chimera software. Using chimera software to predict the hydrogen bond of POLR3A protein structure, it can be seen that the original 767 Cys can form a hydrogen bond with 765 Ser and 764 Gly, but the hydrogen bond cannot be formed after mutation (Fig. 6).

## Discussion

HLD-7 is an autosomal recessive hereditary leukodystrophy associated with *POLR3A* mutation^16^. *POLR3A* is located on the q22.3 of chromosome 10 and consists of 31 exons, encoding Pol III catalytic subunit A (POLR3A) protein^14^. POLR3A is essential for normal nucleolus function, ribosome assembly, and protein translation^17,18^. POLR3A interacts with multiple subunits of Pol III. POLR3A pathogenic mutants can directly interfere with the ability to bind DNA, alter the catalytic cleft structure, and interfere with the protein interactions between POLR3A and other Pol III subunits, thereby impairing the normal assembly of Pol III^19^.

Interestingly, the effect of *POLR3A* mutations on protein expression seems to be variable in different studies. Previous studies have found that the level of POLR3A protein decreased in fibroblasts derived from patients with 4H syndrome, and the level of POLR3A protein in cortical and cerebral white matter of individuals with 4H syndrome decreased, with a more significant decrease in cerebral white matter^15^. Reduced levels of POLR3A protein were also observed in HEK293 clonal cell lines and MO3.13 cell lines carrying the *POLR3A* p.M852V mutation^20^. However, in a study of the effects of *POLR3A* mutations on mRNA and proteins in fibroblasts from patients with Wiedemann-Rautenstrauch syndrome, *POLR3A* mutations resulted in a decrease in wild-type *POLR3A* mRNA and POLR3A protein expression, while the expression of mutant proteins increased sharply ^18^. In addition, in the POLR3-HLD mouse model with the import of the *POLR3A* c.2015G > A (p.Gly672Glu) mutation, the levels of the POLR3A protein in the mutant mice and the major protein components of myelin in the mouse cerebellum (myelin basic protein (MBP), proteolipid protein, and myelin-associated glycoprotein) were comparable to those of wild-type mice, and the myelin formation of mutant mice was not significantly impaired ^21^. In fibroblasts from a patient carrying a compound heterozygous mutation in *POLR3A* c.3721G > A (p.Val1241Met) and the splicing region c.1771-6C > G, quantitative real-time PCR assays showed that the RNA expression level of Pol III target genes (HNRNPH2, UBB, LTF, and HSP90AA1) decreased and overexpression of wild-type POLR3A protein rescued the basic expression of Pol III target genes or reached higher levels, while overexpression of POLR3A protein with p.Val1241Met mutation did not rescue their expression or even appeared at lower levels^16^. This not only confirms the pathogenicity of *POLR3A* p.Val1241Met, but also suggests that the mutant POLR3A protein is functionally weaker than the wild type, and its elevated expression may even cause alteration or loss of Pol III function.

The transcriptional activity of Pol III is closely related to the regulation of cell growth, cell cycle, and cell differentiation processes^22^. Two hypotheses exist regarding potential possible mechanisms for POLR3-HLD: one is that mutations in the gene encoding the Pol III subunit result in reduced levels of tRNA and/or other NC-RNAs that are critical to myelin formation^23^. Oligodendrocytes are myelin-forming cells of the CNS. During myelin development or remyelination, oligodendrocytes need to produce large amounts of proteins to mature, and disruption of tRNA transcription can cause dysregulation of global protein translation during the critical phase of myelin development, ultimately leading to abnormal myelin formation^5,24^. The second hypothesis suggests that reduced Pol III function can lead to reduced levels of specific Pol III transcripts involved in transcription, RNA processing, and translation, thus affecting the development and function of oligodendrocytes and/or neurons^23^.

Alterations in the expression of POLR3A-regulated genes and ribosomal transcripts may affect ribosome production, protein synthesis, and the regulatory processes of RNA polymerases I and II^18^. 5S rRNA is the smallest RNA component of the ribosome, and the cytoplasmic ribosomes of all species contain highly conserved 5S rRNA^25^. The presence of 5S rRNA, a component of the large ribosomal subunit, is extremely important for the normal function of the ribosome^26^. 7SK RNA is a nuclear transcript produced by Pol III, which plays a crucial role in the transcriptional regulation of RNA polymerase II through binding to the transcriptional elongation factor P-TEFb^27^. It has been suggested that 7SK RNA is a major regulator of neuronal development and function^28^. Expression levels of tRNA^imet^, 5S rRNA, 7SK RNA, and 7SL RNA were found to be decreased in fibroblasts from patients with HLD affected by the *POLR3K* mutation^26^. In contrast, blood RNA analysis in patients with striatal involvement phenotype due to *POLR3A* mutation showed an overall decrease in tRNA abundance and an increase in 5S rRNA and 7SK RNA levels^29^. In a study in which endogenous *POLR3A* c.2554A > G (p.M852V) was introduced into HEK293 cells by CRISPR-Cas9 technique to obtain a homozygous mutant clone and two compound heterozygous clones with null allele, the *POLR3A* mRNA levels of the compound heterozygote with p.M852V mutation were decreased, but the *POLR3A* mRNA level of the homozygote with p.M852V mutation was not decreased; precursor tRNA levels were reduced as a whole in all three mutant cells^20^. In our study p.Cys767Phe mutant resulted in increased expression of mutant POLR3A protein. To further analyze the role of the p.Cys767Phe mutant in transcriptional control, we used RT-qPCR to detect the expression of Pol III transcripts. We observed that both tRNA-Leu-CAA and 18S rRNA levels were decreased after the *POLR3A* p.Cys767Phe mutation compared to the wild type. Considering that Pol III is central to tRNA production, changes in tRNA may be the main pathogenic mechanism of the *POLR3A* mutant^15^, and the decrease of 18S rRNA level suggests that this mutant may have an impact on the transcriptional regulation of RNA polymerase I. In addition, 5S rRNA levels were elevated after the p.Cys767Phe mutation compared to the wild type. Although the levels of the transcriptional regulator 7SK RNA after the p.Cys767Phe mutation were not significantly different from those of the wild type group, there was an overall trend of increased levels of 7SK RNA compared to the control group. The increased expression of Pol III-specific transcripts may be a compensatory response to the decrease of tRNA level and preferential recruitment of Pol III to their promoters^29^.

This study also observed that the *POLR3A* p.Cys767Phe mutation led to a decrease in the level of BC200 RNA. *BC200* is a long non-coding RNA that plays an important regulatory role in the translation process of human neuronal cells^30^. BC200 RNA was the most downregulated Pol III transcript in several datasets of *POLR3A* p.M852V mutant cells; moreover, BC200 RNA was also decreased in fibroblasts from patients with POLR3-HLD and in the MO3.13 cell line of oligodendrocytes carrying the *POLR3A* c.2554A > G (p.M852V) mutation^20^. The above results indicated that BC200 RNA may be particularly sensitive to Pol III functional abnormalities^20^, and may play an essential role in the pathogenic mechanism of POLR3-HLD. However, considering that BC200 RNA was overexpressed in a variety of tumor cells and was critical to cancer cell survival and proliferation^31^, and MO3.13 cell lines were established from tumors, it cannot be excluded that BC200 RNA expression and function are unique to this cell line^20,32^. In addition, the POLR3A p.Cys767Phe mutation also caused the decrease of *MBP* mRNA expression in this study. MBP is the second most abundant protein in the myelin sheath of the CNS and is an adhesion agent in the formation of multilayered dense myelin sheaths of the mammalian CNS, playing an important role in myelin formation^33^. MO3.13 cell lines expressing oligodendrocyte precursor cells characteristics could differentiate into a more mature oligodendrocyte phenotype with enhanced *MBP* expression^34^. After the differentiation of MO3.13 cells, the mRNA level of the *MBP* gene in *POLR3A* p.M852V mutant cells was significantly lower than that in wild-type cells ^20^. This suggested that the *POLR3A* mutation could impair the expression of MBP, an important marker for mature oligodendrocytes. Indeed, the functional consequences of various mutations in the genes encoding Pol III subunits are unknown, and how Pol III mutations cause diseases that are mainly confined to the CNS and a few other tissues remains a mystery^23^. Because differences in the affected CNS cell types may result in different clinical phenotypes, Pol III dysfunction may affect different cell types differently, leading to a diversity of disease mechanisms^23^.

From a clinical perspective, there is also heterogeneity in the clinical phenotype caused by POLR3-HLD, which can involve both the neurological and non-neurological systems. Typical neurological involvement can present with neurodevelopmental delays such as learning difficulties and intellectual disability, and can also result in signs and symptoms of cerebellar, pyramidal, and extrapyramidal system involvement such as ataxia, tremor, dysarthria, spasticity, and dystonia^35^. Non-neurological features usually include dental abnormalities (absent or delayed eruption of teeth); endocrine abnormalities (delayed, halted, or absent puberty and short stature); and ocular abnormalities (myopia, optic atrophy, and cataracts)^7^. POLR3-HLD often occurs in infancy or childhood, and the earlier the age of onset, the more severe the clinical phenotype is likely to be^36^. Compared with *POLR3B* mutations, *POLR3A* mutations usually lead to more serious clinical phenotypes and shorter life expectancy for patients^37^. Craniocerebral MRI has high sensitivity in the diagnosis of white matter abnormalities in the CNS. The typical MRI presentation of POLR3-HLD was diffuse hypomyelination with variable T1 signal intensity (low, high, or equal signal), T2 high signal in the white matter of the brain, and relative T2 hypointense signal in specific structures such as the anterolateral nuclei of the thalami, dentate nucleus, globus pallidus, pyramidal tracts of the posterior limb of the internal capsule, and optic radiations; cerebellar atrophy; and thinning of the corpus callosum^5,7,19,38^. Compared to *POLR3B* mutations, hypomyelination was more obvious on MRI in patients with *POLR3A* mutations, whereas the changes in the cerebellar hemispheres and vermis were significantly milder^35,39^. *POLR3A* mutation can lead to gradually obvious cerebellar signs, but cases of cerebellar atrophy not observed by MRI have been previously reported^40^. It should be noted that diffuse hypomyelination is not a necessary imaging feature of POLR3-related disease, and patients with *POLR3A* or *POLR3B* mutations can present without hypomyelination^41,42^. However, the simultaneous appearance of the above MRI manifestations is a unique imaging feature of POLR3-HLD^43^.

Due to the high phenotypic heterogeneity of POLR3A mutations, different families carrying the same mutation may result in different phenotypes^4^. However, distinct intrafamilial variation is relatively rare ^37^. Interestingly, members of the same family in our study carried the same variant, but there was significant clinical heterogeneity between them. All the cases in this study had delayed puberty and abnormal teeth, and the first mental symptoms were prominent, with cognitive impairment. Motor function was also affected in the older brothers of the proband. Among them, the proband was characterized by cognitive dysfunction and endocrine abnormality, puberty development was delayed, and the development of breasts and pubes stagnated at Tanner Stage II. After receiving long-term hormone replacement therapy, repeated monitoring of E2, FSH, LH, and P4 remained at low levels. Combined with the results of the GnRH stimulation test and craniocerebral MRI, the gonadotropin deficiency of the proband was considered to be at the pituitary level. Craniocerebral MRI of the proband showed brain atrophy, atrophic degeneration of the white matter of the brain, and atrophy and thinning of the corpus callosum, but no obvious symptoms of ataxia or motor impairment. At the onset of POLR3-HLD, it may only show cognitive dysfunction, but motor symptoms do not exist^35^. Unlike the proband, her three old brothers have varying degrees of ataxia, dystonia, or dysarthria in addition to cognitive impairment, and have a more rapid progression of impaired motor function. In addition, the proband had microalbuminuria and her father had the phenotype of proteinuria, nephritis, and uremia, while the other members of the family did not have renal complications. *POLR3A* mutations are associated with WRS, and a case of fatal hyperkalemic renal failure in a three-day-old male infant with features of WRS has been reported, but whether WRS is associated with structural renal anomalies requires more evidence to confirm^44^. Regrettably, we currently also have no evidence to suggest a correlation between the renal phenotype of this family and the *POLR3A* c.2300G > T mutant.

As the diagnosis of POLR3-HLD is a combination of typical clinical presentation, brain MRI features, and pathogenic gene testing, the complexity of the clinical phenotype may pose a challenge for early diagnosis^45^. Dental developmental abnormalities and hypogonadotropic hypogonadism are not prevalent in POLR3-HLD, and in the absence of dental or endocrine abnormalities, brain MRI may better assist in the diagnosis of the disease^37,38^. When patients have corresponding symptoms or signs, and there are diffuse hypomyelination and cerebellar atrophy on craniocerebral imaging, clinicians should highly doubt the possibility of POLR3-HLD and consider the sequencing of *POLR3A* and *POLR3B* genes in time.

In conclusion, HLD-7 is a hereditary hypomyelinating leukodystrophy and its clinical features may differ greatly. As the disease can involve multiple systems, its diagnosis and management need multidisciplinary comprehensive treatment. The intrafamilial variation in this study not only increased the research value of the family to some extent, but also enriched the clinical phenotypic spectrum of POLR3-HLD disease. Although mutants causing POLR3-HLD may affect the normal assembly or biogenesis of Pol III, the functional mechanisms of how these mutations lead to myelin formation deficiency remains to be investigated. In this study, we found that wild-type POLR3A protein overexpression significantly enhanced Pol III transcription of 5S rRNA, tRNA Leu-CAA; however, the increased overexpression of the p.Cys767Phe mutant POLR3A protein compared with the wild-type protein overexpression did not cause the expected further enhancement of Pol III function, but rather frustrated Pol III transcriptional function, suggesting an abnormal function of the mutant POLR3A protein. This study initially confirmed the pathogenicity of the *POLR3A* p.Cys767Phe mutation, which will provide a reference for further studies on the pathogenic mechanism of POLR3-HLD in the future.

## Supplementary Information

Supplementary Information.

## Data Availability

The datasets generated and/or analysed during the current study are available in the ClinVAR repository, and the accession number is SCV003928000 (https://www.ncbi.nlm.nih.gov/clinvar/variation/1473885/).

## References

[1] Thiffault, I. *et al.* Recessive mutations in POLR1C cause a leukodystrophy by impairing biogenesis of RNA polymerase III. *Nat. Commun.* **6**, 7623. 10.1038/ncomms8623 (2015). 26151409 10.1038/ncomms8623 PMC4506509

[2] Schiffmann, R. & van der Knaap, M. S. Invited article: An MRI-based approach to the diagnosis of white matter disorders. *Neurology* **72**, 750–759. 10.1212/01.wnl.0000343049.00540.c8 (2009). 19237705 10.1212/01.wnl.0000343049.00540.c8 PMC2677542

[3] Al Yazidi, G. *et al.* Dystonia in RNA polymerase III-related leukodystrophy. *Mov. Disord. Clin. Pract.* **6**, 155–159. 10.1002/mdc3.12715 (2019). 30838315 10.1002/mdc3.12715 PMC6384176

[4] Di Donato, I. *et al.* POLR3A variants in hereditary spastic paraparesis and ataxia: Clinical, genetic, and neuroradiological findings in a cohort of Italian patients. *Neurol. Sci.* **43**, 1071–1077. 10.1007/s10072-021-05462-1 (2022). 34296356 10.1007/s10072-021-05462-1 PMC8789690

[5] Perrier, S., Michell-Robinson, M. A. & Bernard, G. POLR3-related leukodystrophy: Exploring potential therapeutic approaches. *Front. Cell. Neurosci.* **14**, 631802. 10.3389/fncel.2020.631802 (2020). 33633543 10.3389/fncel.2020.631802 PMC7902007

[6] Dieci, G., Fiorino, G., Castelnuovo, M., Teichmann, M. & Pagano, A. The expanding RNA polymerase III transcriptome. *Trends Genet.* **23**, 614–622. 10.1016/j.tig.2007.09.001 (2007). 17977614 10.1016/j.tig.2007.09.001

[7] Thomas, A. & Thomas, A. K. POLR3-related Leukodystrophy. *J. Clin. Imaging Sci.* **9**, 45. 10.25259/JCIS_116_2019 (2019). 31768296 10.25259/JCIS_116_2019 PMC6826334

[8] Sosinska-Zawierucha, P., Zawierucha, P., Breborowicz, A. & Barciszewski, J. Prediction of secondary and tertiary structures of human BC200 RNA (BCYRN1) based on experimental and bioinformatic cross-validation. *Biochem. J.* **475**, 2727–2748. 10.1042/BCJ20180239 (2018). 30072491 10.1042/BCJ20180239

[9] Muslimov, I. A. *et al.* Autoimmune RNA dysregulation and seizures: Therapeutic prospects in neuropsychiatric lupus. *Life Sci. Alliance* **5**, e202201496. 10.26508/lsa.202201496 (2022). 36229064 10.26508/lsa.202201496 PMC9559755

[10] Borck, G. *et al.* BRF1 mutations alter RNA polymerase III-dependent transcription and cause neurodevelopmental anomalies. *Genome Res.* **25**, 155–166. 10.1101/gr.176925.114 (2015). 25561519 10.1101/gr.176925.114 PMC4315290

[11] Mendes, M. I. *et al.* Bi-allelic mutations in EPRS, encoding the glutamyl-prolyl-aminoacyl-tRNA synthetase, cause a hypomyelinating leukodystrophy. *Am. J. Hum. Genet.* **102**, 676–684. 10.1016/j.ajhg.2018.02.011 (2018). 29576217 10.1016/j.ajhg.2018.02.011 PMC5985283

[12] Nakayama, T. *et al.* Deficient activity of alanyl-tRNA synthetase underlies an autosomal recessive syndrome of progressive microcephaly, hypomyelination, and epileptic encephalopathy. *Hum. Mutat.* **38**, 1348–1354. 10.1002/humu.23250 (2017). 28493438 10.1002/humu.23250 PMC5599341

[13] Schaffer, A. E. *et al.* CLP1 founder mutation links tRNA splicing and maturation to cerebellar development and neurodegeneration. *Cell* **157**, 651–663. 10.1016/j.cell.2014.03.049 (2014). 24766810 10.1016/j.cell.2014.03.049 PMC4128918

[14] Musumeci, A. *et al.* Identification of a novel missense mutation of POLR3A gene in a cohort of sicilian patients with leukodystrophy. *Biomedicines* **10**, 2276. 10.3390/biomedicines10092276 (2022). 36140376 10.3390/biomedicines10092276 PMC9496502

[15] Bernard, G. *et al.* Mutations of POLR3A encoding a catalytic subunit of RNA polymerase Pol III cause a recessive hypomyelinating leukodystrophy. *Am. J. Hum. Genet.* **89**, 415–423. 10.1016/j.ajhg.2011.07.014 (2011). 21855841 10.1016/j.ajhg.2011.07.014 PMC3169829

[16] Zanette, V. *et al.* Neurodevelopmental regression, severe generalized dystonia, and metabolic acidosis caused by POLR3A mutations. *Neurol. Genet.* **6**, e521. 10.1212/NXG.0000000000000521 (2020). 33134517 10.1212/NXG.0000000000000521 PMC7577545

[17] Tiku, V. & Antebi, A. Nucleolar function in lifespan regulation. *Trends Cell Biol.* **28**, 662–672. 10.1016/j.tcb.2018.03.007 (2018). 29779866 10.1016/j.tcb.2018.03.007

[18] Baez-Becerra, C. T. *et al.* Nucleolar disruption, activation of P53 and premature senescence in POLR3A-mutated Wiedemann-Rautenstrauch syndrome fibroblasts. *Mech. Ageing Dev.* **192**, 111360. 10.1016/j.mad.2020.111360 (2020). 32976914 10.1016/j.mad.2020.111360

[19] Gutierrez, M. *et al.* Large exonic deletions in POLR3B gene cause POLR3-related leukodystrophy. *Orphanet J. Rare Dis.* **10**, 69. 10.1186/s13023-015-0279-9 (2015). 26045207 10.1186/s13023-015-0279-9 PMC4520020

[20] Choquet, K. *et al.* Leukodystrophy-associated POLR3A mutations down-regulate the RNA polymerase III transcript and important regulatory RNA BC200. *J. Biol. Chem.* **294**, 7445–7459. 10.1074/jbc.RA118.006271 (2019). 30898877 10.1074/jbc.RA118.006271 PMC6509492

[21] Choquet, K. *et al.* Absence of neurological abnormalities in mice homozygous for the Polr3a G672E hypomyelinating leukodystrophy mutation. *Mol. Brain* **10**, 13. 10.1186/s13041-017-0294-y (2017). 28407788 10.1186/s13041-017-0294-y PMC5391615

[22] Dumay-Odelot, H., Durrieu-Gaillard, S., Da Silva, D., Roeder, R. G. & Teichmann, M. Cell growth- and differentiation-dependent regulation of RNA polymerase III transcription. *Cell Cycle* **9**, 3687–3699. 10.4161/cc.9.18.13203 (2010). 20890107 10.4161/cc.9.18.13203 PMC3047797

[23] Lata, E. *et al.* RNA polymerase III subunit mutations in genetic diseases. *Front. Mol. Biosci.* **8**, 696438. 10.3389/fmolb.2021.696438 (2021). 34395528 10.3389/fmolb.2021.696438 PMC8362101

[24] Elbaz, B. & Popko, B. Molecular control of oligodendrocyte development. *Trends Neurosci.* **42**, 263–277. 10.1016/j.tins.2019.01.002 (2019). 30770136 10.1016/j.tins.2019.01.002 PMC7397568

[25] Huang, S. *et al.* Ribosome engineering reveals the importance of 5S rRNA autonomy for ribosome assembly. *Nat. Commun.* **11**, 2900. 10.1038/s41467-020-16694-8 (2020). 32518240 10.1038/s41467-020-16694-8 PMC7283268

[26] Dorboz, I. *et al.* Mutation in POLR3K causes hypomyelinating leukodystrophy and abnormal ribosomal RNA regulation. *Neurol. Genet.* **4**, e289. 10.1212/NXG.0000000000000289 (2018). 30584594 10.1212/NXG.0000000000000289 PMC6283457

[27] Roder, K., Stirnemann, G., Dock-Bregeon, A. C., Wales, D. J. & Pasquali, S. Structural transitions in the RNA 7SK 5’ hairpin and their effect on HEXIM binding. *Nucleic Acids Res.* **48**, 373–389. 10.1093/nar/gkz1071 (2020). 31732748 10.1093/nar/gkz1071 PMC7145557

[28] Briese, M. & Sendtner, M. Keeping the balance: The noncoding RNA 7SK as a master regulator for neuron development and function. *Bioessays* **43**, e2100092. 10.1002/bies.202100092 (2021). 34050960 10.1002/bies.202100092

[29] Azmanov, D. N. *et al.* Transcriptome-wide effects of a POLR3A gene mutation in patients with an unusual phenotype of striatal involvement. *Hum. Mol. Genet.* **25**, 4302–4314. 10.1093/hmg/ddw263 (2016). 27506977 10.1093/hmg/ddw263

[30] Yeganeh, M. & Hernandez, N. RNA polymerase III transcription as a disease factor. *Genes Dev.* **34**, 865–882. 10.1101/gad.333989.119 (2020). 32611613 10.1101/gad.333989.119 PMC7328520

[31] Booy, E. P., McRae, E. K., Koul, A., Lin, F. & McKenna, S. A. The long non-coding RNA BC200 (BCYRN1) is critical for cancer cell survival and proliferation. *Mol. Cancer* **16**, 109. 10.1186/s12943-017-0679-7 (2017). 28651607 10.1186/s12943-017-0679-7 PMC5483959

[32] McLaurin, J., Trudel, G. C., Shaw, I. T., Antel, J. P. & Cashman, N. R. A human glial hybrid cell line differentially expressing genes subserving oligodendrocyte and astrocyte phenotype. *J. Neurobiol.* **26**, 283–293. 10.1002/neu.480260212 (1995). 7707048 10.1002/neu.480260212

[33] Boggs, J. M. Myelin basic protein: A multifunctional protein. *Cell. Mol. Life Sci.* **63**, 1945–1961. 10.1007/s00018-006-6094-7 (2006). 16794783 10.1007/s00018-006-6094-7 PMC11136439

[34] Voccoli, V., Tonazzini, I., Signore, G., Caleo, M. & Cecchini, M. Role of extracellular calcium and mitochondrial oxygen species in psychosine-induced oligodendrocyte cell death. *Cell Death Dis.* **5**, e1529. 10.1038/cddis.2014.483 (2014). 25412308 10.1038/cddis.2014.483 PMC4260741

[35] Campopiano, R. *et al.* A novel POLR3A genotype leads to leukodystrophy type-7 in two siblings with unusually late age of onset. *BMC Neurol.* **20**, 258. 10.1186/s12883-020-01835-9 (2020). 32600288 10.1186/s12883-020-01835-9 PMC7322863

[36] Tewari, V. V. *et al.* A novel homozygous mutation in POLR3A gene causing 4H syndrome: A case report. *BMC Pediatr.* **18**, 126. 10.1186/s12887-018-1108-9 (2018). 29618326 10.1186/s12887-018-1108-9 PMC5883641

[37] Wolf, N. I. *et al.* Clinical spectrum of 4H leukodystrophy caused by POLR3A and POLR3B mutations. *Neurology* **83**, 1898–1905. 10.1212/WNL.0000000000001002 (2014). 25339210 10.1212/WNL.0000000000001002 PMC4248461

[38] Steenweg, M. E. *et al.* Magnetic resonance imaging pattern recognition in hypomyelinating disorders. *Brain* **133**, 2971–2982. 10.1093/brain/awq257 (2010). 20881161 10.1093/brain/awq257 PMC3589901

[39] Vrij-van den Bos, S. *et al.* 4H leukodystrophy: A brain magnetic resonance imaging scoring system. *Neuropediatrics* **48**, 152–160. 10.1055/s-0037-1599141 (2017). 28561206 10.1055/s-0037-1599141

[40] Wu, S. *et al.* Novel mutations of the POLR3A gene caused POLR3-related leukodystrophy in a Chinese family: A case report. *BMC Pediatr.* **19**, 289. 10.1186/s12887-019-1656-7 (2019). 31438894 10.1186/s12887-019-1656-7 PMC6704677

[41] La Piana, R. *et al.* Diffuse hypomyelination is not obligate for POLR3-related disorders. *Neurology* **86**, 1622–1626. 10.1212/WNL.0000000000002612 (2016). 27029625 10.1212/WNL.0000000000002612 PMC4844237

[42] Harting, I. *et al.* POLR3A variants with striatal involvement and extrapyramidal movement disorder. *Neurogenetics* **21**, 121–133. 10.1007/s10048-019-00602-4 (2020). 31940116 10.1007/s10048-019-00602-4 PMC7064625

[43] La Piana, R. *et al.* Brain magnetic resonance imaging (MRI) pattern recognition in Pol III-related leukodystrophies. *J. Child Neurol.* **29**, 214–220. 10.1177/0883073813503902 (2014). 24105487 10.1177/0883073813503902

[44] Ghamry, M. A., Salah, R., Galal, E. I., Henin, S. & Dobs, M. A case of Wiedemann-Rautenstrauch syndrome with fatal hyperkalemic renal faliure. *Cureus* **14**, e29320. 10.7759/cureus.29320 (2022). 36159344 10.7759/cureus.29320 PMC9484294

[45] Billington, E., Bernard, G., Gibson, W. & Corenblum, B. Endocrine aspects of 4H leukodystrophy: A case report and review of the literature. *Case Rep. Endocrinol.* **2015**, 314594. 10.1155/2015/314594 (2015). 26113998 10.1155/2015/314594 PMC4465690

