# Author Correction: Clinical phenotype and genetic function analysis of a family with hypomyelinating leukodystrophy-7 caused by *POLR3A* mutation

**DOI:** 10.1038/s41598-024-72293-3

**Published:** 2024-09-10

**Authors:** Dan-dan Ruan, Xing-lin Ruan, Ruo‑li Wang, Xin-fu Lin, Yan-ping Zhang, Bin Lin, Shi-jie Li, Min Wu, Qian Chen, Jian-hui Zhang, Qiong Cheng, Yi-wu Zhang, Fan Lin, Jie-wei Luo, Zheng Zheng, Yun-fei Li

**Affiliations:** 1 Department of Traditional Chinese Medicine, Shengli Clinical Medical College of Fujian Medical University, Fujian Provincial Hospital, Fuzhou, China; 2 Department of Neurology, Fujian Medical University Union Hospital, Fuzhou, 350001 China; 3 Fujian Provincial Key Laboratory of Emergency Medicine, Fujian Provincial Institute of Emergency Medicine, Fujian Emergency Medical Center, Fuzhou, 350001 China; 4 Pediatrics Department, Fujian Provincial Hospital, Fuzhou, 350001 China; 5 Department of Neurology, Fujian Provincial Hospital, Fuzhou, 350001 China; 6 Department of Neurology, Youxi County General Hospital, Sanming, 365100 China; 7 Department of Geriatric Medicine, Fujian Provincial Center for Geriatrics, Fujian Provincial Hospital, Fuzhou, 350001 China

Correction to: *Scientific Reports* 10.1038/s41598-024-58452-6, published online 01 April 2024

The original version of this Article contained an error in Affiliation 1, which was incorrectly given as ‘Department of Traditional Chinese Medicine, Fujian Provincial Hospital, Shengli Clinical Medical College of Fujian Medical University, Fuzhou, 350001, China’. The correct affiliation is listed below:

Department of Traditional Chinese Medicine, Shengli Clinical Medical College of Fujian Medical University, Fujian Provincial Hospital, Fuzhou, China.

The original Article has been corrected.

