# Supplementary material for: Clinical phenotype and genetic function analysis of a family with hypomyelinating leukodystrophy-7 caused by POLR3A mutation
Source: Sci Rep. 2024 Apr 1;14:7638. doi: 10.1038/s41598-024-58452-6 (PMC10985069; doi:10.1038/s41598-024-58452-6)
Supplement: Supplementary file 1 — Supplementary Information. [file 41598_2024_58452_MOESM1_ESM.pdf]

Clinical phenotype and genetic function analysis of a family with hypomyelinating leukodystrophy-7 caused by *POLR3A* mutation

Dan-dan Ruan[1†], Xing-lin Ruan[2†], Ruo-li Wang [1, 3†], Xin-fu Lin[1, 4†], Yan-ping Zhang[1], Bin Lin[1], Shi-jie Li[1], Min Wu[1], Qian Chen[1], Jian-hui Zhang[1], Qiong Cheng[1,5], Yi-wu Zhang [6], Fan Lin[1,7*], Jie-wei Luo[1,8*], Zheng Zheng[1,5*], Yun-fei Li[1,5*]

Supplementary Table S1 Detailed results of electromyography of the proband (IV6)

**Motor nerve conduction study**				
Nerve	Lat (ms)	Amp (mV)	CV (m/s)	F-M Lat (ms)
Ulnar nerve, motor, left				
wrist-abductor digit minimi muscle	1.69	13.9		
elbow-wrist	6.48	13.2	54.3	
Ulnar nerve, motor, right				
wrist-abductor digit minimi muscle	2.00	15.5		
elbow-wrist	6.65	14.6	64.5	
Median nerve, motor, left				
wrist-abductor pollicis brevis muscle	3.04	12.4		21.1
elbow-wrist	6.85	12.0	60.4	
Median nerve, motor, right				
wrist-abductor pollicis brevis muscle	2.63	16.2		19.1
elbow-wrist	6.60	14.6	55.4	
Tibial nerve, motor, left				
malleolus-abductor hallucis muscle	4.31	12.1		
Tibial nerve, motor, right				
malleolus-abductor hallucis muscle	3.50	13.7		
Common peroneal nerve, motor, left				
malleolus-extensor digitorum brevis muscle	3.56	8.8		
fibular head- malleolus	10.1	7.6	49.7	
Common peroneal nerve, motor, right				
malleolus-extensor digitorum brevis muscle	3.14	8.7		
fibular head- malleolus	10.6	7.6	40.2	
**Sensory nerve conduction study**				
Nerve	Peak Lat (ms)	Amp (uV)	CV (m/s)	
Ulnar nerve, feeling, left				
finger V- wrist	2.00	15.0	62.5	
Ulnar nerve, feeling, right				
finger V- wrist	2.08	16.5	57.7	

Median nerve, feeling, left						
finger I-wrist	1.66	40.6	66.3			
finger III-wrist	2.17	33.9	71.4			
Median nerve, feeling, right						
finger I-wrist	1.67	40.7	59.9			
finger III-wrist	2.13	28.9	63.4			
Tibial nerve, feeling, left						
toe I-malleolus	3.92	1.44	51.0			
Tibial nerve, feeling, right						
toe I-malleolus	4.24	2.8	44.8			
Common peroneal nerve, feeling, left						
fibula inferior-malleolus	4.63	3.3	64.8			
Common peroneal nerve, feeling, right						
fibula inferior-malleolus	5.17	3.4	58.0			
Sural nerve, feeling, right						
crus medial-lateral malleolus	2.67	13.0	50.6			
Sural nerve, feeling, left						
crus medial-lateral malleolus	1.82	14.1	63.2			
**F-wave**						
	M-Lat (ms)	F-Lat (mean, ms)	F-M Lat (ms)	Amp (uV)	F-wave occurrence rate (%)	CV (m/s)
Ulnar nerve F-wave left						
wrist-abductor digit minimi muscle	1.50	26.1	24.6	256	100	63.7
Ulnar nerve F-wave right						
wrist-abductor digit minimi muscle	1.00	25.8	24.8	302	100	59.0
Median nerve F-wave left						
wrist-abductor pollicis brevis muscle	2.1	24.5	22.4	271	100	63.7
Median nerve F-wave right						
wrist-abductor pollicis brevis muscle	2.4	22.1	19.7	373	100	70.7
Tibial nerve F-wave left						
medial malleolus-abductor hallucis muscle	4.1	48.8	44.8	175	100	--
Tibial nerve F-wave right						
medial malleolus-abductor hallucis muscle	3.4	46.0	42,.6	408	100	--

Note: Lat, Latency; Amp, Amplitude; CV, Conduction velocity

Supplementary Table S2 Detailed results of somatosensory evoked potentials of the proband(IV6)

**Upper limbs**					
			Latency		
			Left (ms)	Right (ms)	Side Diff (ms)
Mean	C4'-Fz/C3'-Fz	N20	19.5	18.5	1.00
	C7-Fz	N13	13.6	13.6	0
	Erb'	N9	9.2	9.3	0.10
**Lower limbs**					
			Latency		
			Left (ms)	Right (ms)	Side Diff (ms)
Mean	Cz'-Fpz	N48	50.3(↑)	46.1	4.2
	T12-Sc2	N21	25.7(↑)	21.2	4.5
	pf	N9	7.1	7.0	0.10

Supplementary Fig. S1 Gel diagram of Western Blot

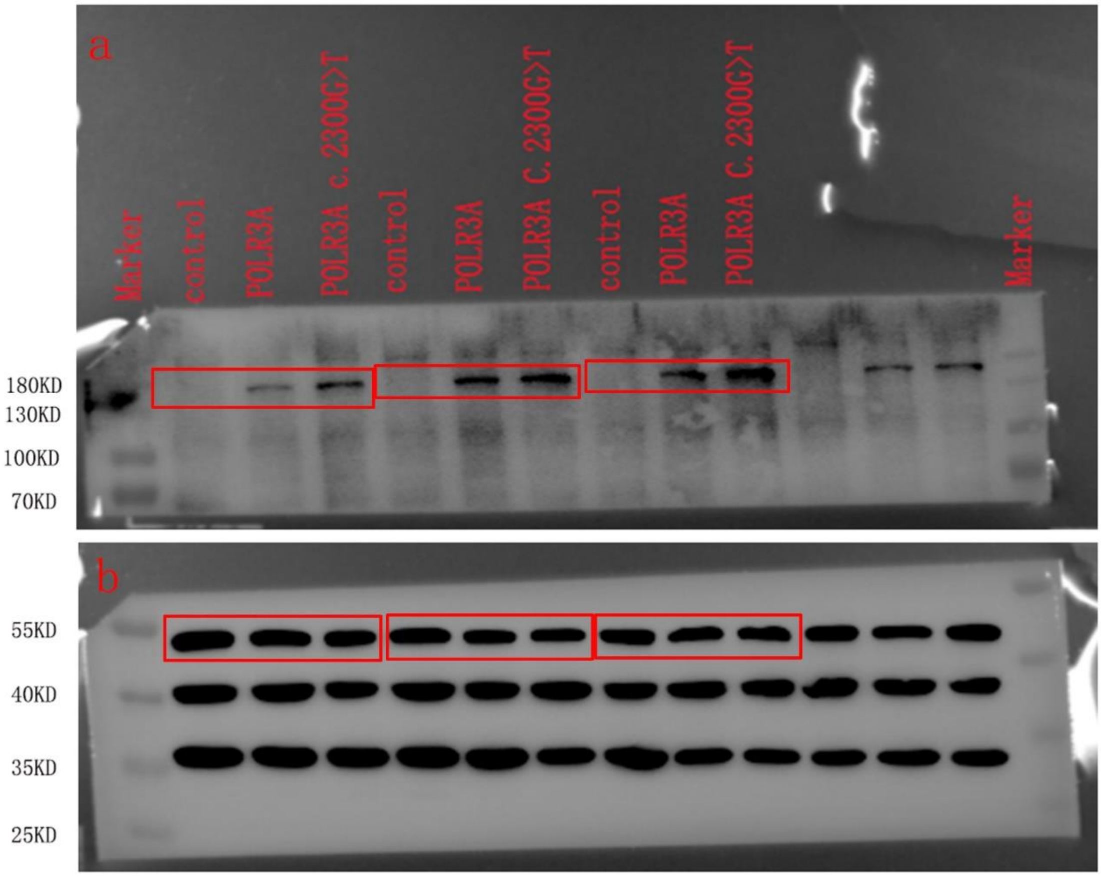

Fig. S1 Gel diagram of Western Blot. a. FLAG merge. b. Loading control merge: α-Tubulin (55 KD), β-Actin (42 KD), and GAPDH (36 KD).
